# Simple Ways to Measure Behavioral Responses of *Drosophila* to Stimuli and Use of These Methods to Characterize a Novel Mutant

**DOI:** 10.1371/journal.pone.0037495

**Published:** 2012-05-23

**Authors:** Lar L. Vang, Alexei V. Medvedev, Julius Adler

**Affiliations:** 1 Departments of Biochemistry and Genetics, University of Wisconsin-Madison, Madison, Wisconsin, United States of America; 2 Department of Microbiology and Immunology, Georgetown University, Georgetown, Washington, D.C., United States of America; Harvard University, United States of America

## Abstract

The behavioral responses of adult *Drosophila* fruit flies to a variety of sensory stimuli – light, volatile and non-volatile chemicals, temperature, humidity, gravity, and sound - have been measured by others previously. Some of those assays are rather complex; a review of them is presented in the Discussion. Our objective here has been to find out how to measure the behavior of adult *Drosophila* fruit flies by methods that are inexpensive and easy to carry out. These new assays have now been used here to characterize a novel mutant that fails to be attracted or repelled by a variety of sensory stimuli even though it is motile.

## Introduction

Organisms respond behaviorally to various stimuli in their environment: for example, light, chemicals, temperature, humidity, gravity, and sound. The sensing of these stimuli is carried out, respectively, by vision, smell of volatile chemicals (olfaction), taste of nonvolatile chemicals (gustation), thermosensors, humidity sensors, gravity sensors, and hearing. The responses to stimuli can be attraction or repulsion, depending on the nature and the intensity of the stimulus.

Recent reports summarize that adult *Drosophila melanogaster* fruit flies are behaviorally responsive to light [1]–[3], chemicals: odors [4]–[7] and tastants [4], [8], [9], humidity [10], temperature [11]–[13], gravity [14]–[17], and sound [14], [18]. There are many methods for assaying these responses by *Drosophila* quantitatively; see descriptions of these in the Discussion. Some of those assays are simple; others are relatively complex or expensive or involve custom-made equipment.

In this report we describe easy assays for measuring behavioral responses to various stimuli in *D. melanogaster*. Also, we present an application of these assays to characterize novel mutants we have isolated [Lar L. Vang, Andrew M. Winter, Leann M. Erlien, Alex R. Kleven, Nathan J. Menninga, Keegan M. Schlittler, Sarah K. Warzon, Robert A. Kreber, and Julius Adler in “*Drosophila* mutants that are motile but fail to respond to stimuli”, manuscript in preparation, 2012].

## Materials and Methods

*D. melanogaster* of the Canton-Special (CS) strain was raised on cornmeal-molasses-agar medium at room temperature (21–23°C) in 12 hours of light and 12 hour of dark. All experiments were performed at room temperature (21–23°C unless otherwise stated) with flies (males together with females unless otherwise stated) that were up to 1.5 weeks old.

### I. Methods to measure responses

#### A. Response to light: phototaxis

**Figure 1:**
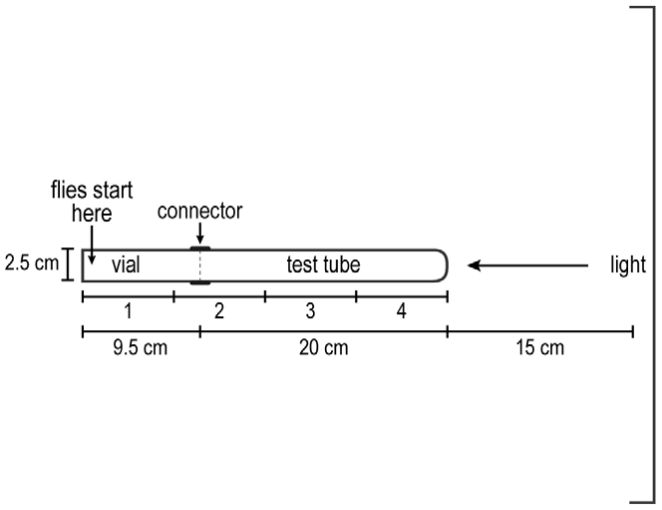
How to measure response to light. Flies start out in the vial and go to the light source. Readings are in 4 parts as indicated. See text.

Increasing light intensity, see Figure 1. The apparatus consisted of a vial (2.5×9.5 cm) (Fisher AS-574) and a test tube (2.5×20 cm) (Fisher 14-925N) put together by a connector (2.5 cm×2.5 cm) (cut from transparent plastic centrifuge tubes 2.5×8.9 cm, Ultra-Clear, Beckman). (In place of this connector, transparent tape could be used.) A visible-light source (2.8 cm×45.2 cm, 15 watt) (Sylvania F15T8/CW/6PK) established a gradient of light that acted as an attractant for the flies, about 3,000 lux at the nearest point of the apparatus and about 700 lux at the furthest point.

In a dark room the vial containing about 20 flies plugged by cotton and the test tube were left separately for 30 minutes. This allowed adaptation of the flies to darkness. The vial was then gently pounded down to place the flies at the end away from the cotton, the cotton was removed, and the vial was attached to the test tube by a connector. This apparatus was horizontal and perpendicular to the horizontal light source 15 cm away. The light was then turned on and a timer was started. The flies were counted every minute for each quarter of the apparatus. In a control, the apparatus was placed 15 cm away from and parallel to the light source.

### B. Response to volatile chemicals: smell chemotaxis

**Figure 2:**
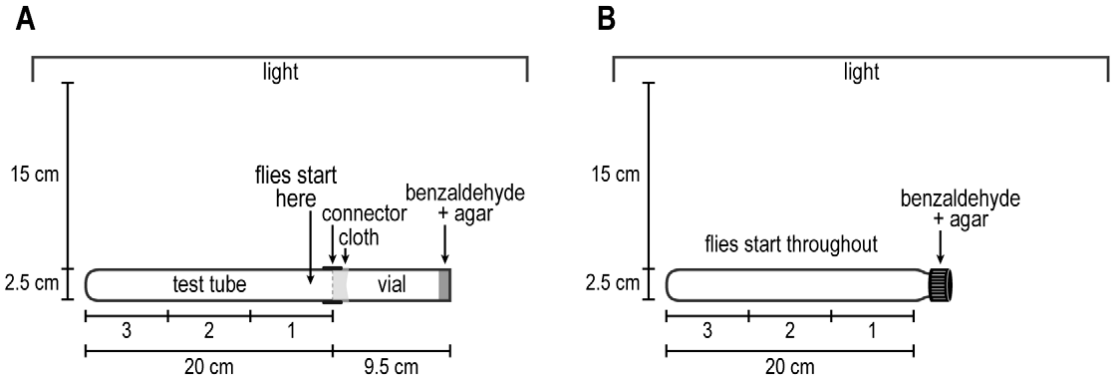
How to measure response to a volatile repellent, benzaldehyde. Method 1: A) Flies start out nearest the repellent and then run away from the repellent. Readings are in 3 parts as indicated. Method 2: B) Flies are randomly distributed at the start and then run away from the repellent. Readings are in 3 parts as indicated. See text.

Volatile repellent, benzaldehyde, see Figures 2A and 2B. The experiment was carried out in a dark room 15 cm away from a turned-on parallel visible-light source, which served for visibility throughout the duration of the experiment. (A lighted room also works but the light must be uniform, which may be difficult to achieve, because non-uniform light is itself a stimulus.) For Method 1 (Figure 2A) about 20 flies were placed into a vial, it was plugged with cotton, then it was placed into the dark room for 30 minutes parallel to the light source to adapt the flies to this level of light. At 15 minutes into incubation 1 ml of 100 mM benzaldehyde dissolved in 1.5% agar was pipetted into another vial, which was then covered with mesh cloth (we used tulle); a connector served to keep the cloth in place. This vial of benzaldehyde was covered with Parafilm and then placed into the dark room. At 30 minutes the flies were gently pounded into a 2.5×20 cm test tube, which was then connected to the benzaldehyde vial by means of the connector, all in the dark room. Then the flies were gently pounded to the cloth. Starting at that time, they were counted every minute in each third of the test tube for 20 minutes. A control without benzaldehyde was also run.

For Method 2 (Figure 2B) the experiment was again carried out in a dark room 15 cm away from a turned-on parallel visible-light source. About 20 flies were placed into a 2.5×20 cm test tube with a screw cap (Fisher 14-930F) and kept in the dark room for 30 minutes parallel to the light source. At that time the flies were randomly distributed. Then the screw cap was gently removed and replaced with a screw cap containing 1 ml of 100 mM benzaldehyde dissolved in 1.5% agar. The number of flies in each third of the test tube was then counted every minute for 10 minutes.

### C. Response to nonvolatile chemicals: taste chemotaxis

**Figure 3:**
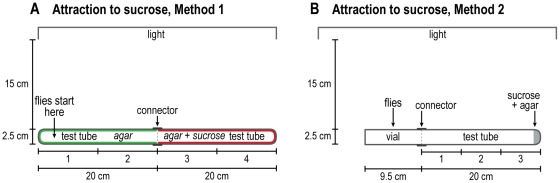
How to measure response to a non-volatile attractant, sucrose. Method 1: A) The red tube indicates agar plus sucrose, the blue tube agar without sucrose. Flies start out at the left end and go to the sucrose. Readings are in 4 parts as indicated. Method 2: B) Flies start out in the vial and go to the sucrose-agar end. Readings are in 3 parts as indicated. See text.

Non-volatile attractant, sucrose, see Figures 3A and 3B. Either the flies were used without starvation, or they were first starved in presence of 1 ml of 1.5% agar for 18 hours to make them hungry. Method 1 (Figure 3A): In one 2.5×20 cm test tube that was microwaved to make warm the entire interior was evenly coated with 3 ml of 1.5% agar, and in another 2.5×20 cm test tube, it was also warmed and coated with 3 ml of 1.5% agar containing 100 mM sucrose. This was done by placing melted agar into the tube and then rotating the tube horizontally, taking care to ensure even coating. The exterior of the tube was then run under cool water until the agar hardened. A vial was coated in the same way with 1 ml of 1.5% agar, about 20 flies were put into it, and it was plugged with cotton. All three were then placed in a dark room for 30 minutes 15 cm away from a turned-on parallel visible-light source. The cotton was then removed, the flies were put into the non-sucrose test tube and pounded down, and the two test tubes were put together with a connector. A timer was then started and the number of flies was scored every minute in each quarter of the test tubes. In a control, both tubes contained only agar, no sucrose.

Method 2 (Figure 3B): In an alternative assay that did not involve coating with agar, 100 mM sucrose in 1 ml of 1.5% agar was placed at the end of a 2.5×20 cm test tube, then the starved flies in a vial were connected to the test tube (after 30 min in the dark as above). A timer was started and the flies in each third of the test tube were scored every minute. In a control the agar contained no sucrose.

**Figure 4:**
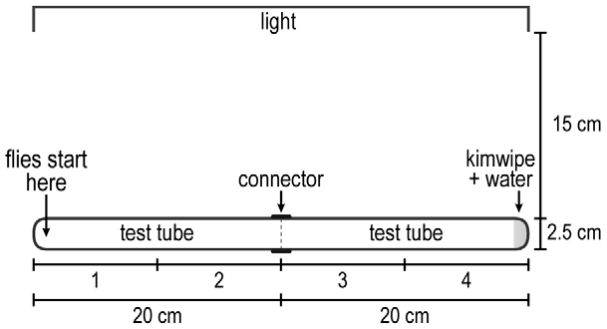
How to measure response to water. Flies start out at the left end and go to the water-Kimwipe end. Readings are in 4 parts as indicated. See text.

Attraction to water, see Figure 4. Flies were deprived of water for 18 hours by placing them in a vial containing sucrose crystals for food. Then about 20 of these flies were put into an empty vial plugged with cotton for 30 minutes in a dark room 15 cm away from a parallel always turned-on visible-light source. A 2.5×20 cm test tube with a connector attached (covered with parafilm) and another 2.5×20 cm test tube with Kimwipe and 0.5 ml distilled water (covered with parafilm) were also placed parallel to the light source. After 30 minutes, the flies were then transferred into the test tube without Kimwipe and pounded down to bring them to its end, then this tube was put together with the test tube that had Kimwipe soaked in distilled water. The number of flies was counted every minute in each quarter. In a control, the same was done except that no water was added to the Kimwipe. (Water of course is volatile but see Results and Discussion.)

**Figure 5:**
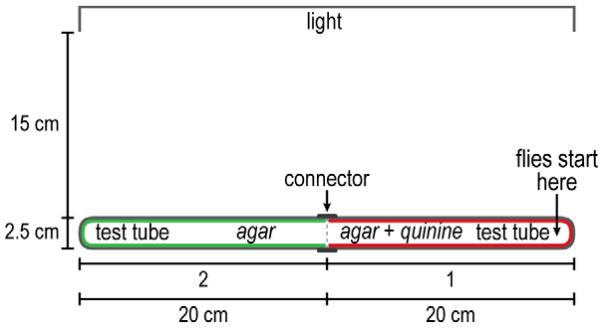
How to measure response to a non-volatile repellent, quinine HCl. The red tube indicates agar plus quinine, the blue tube agar without quinine. Flies start out at the right end and go away from the quinine. Readings are in each of the halves. See text.

Non-volatile repellent, quinine HCl, see Figure 5. The procedure was the same as for sucrose in Figure 3A, including agar throughout, except that the flies were not starved, they started at the rounded end of the 100 mM quinine HCl half, and the number of flies in each half was scored. In a control, no quinine was used.

#### D. Response to humidity: hygrotaxis

**Figure 6:**
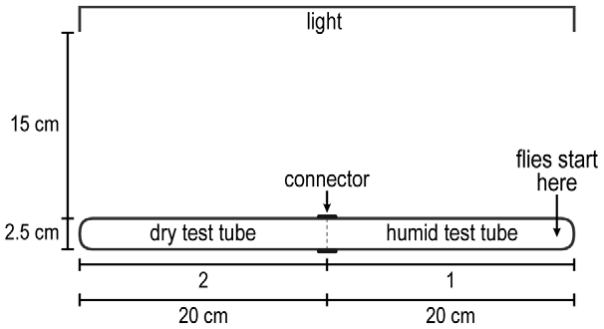
How to measure response to high humidity. Flies start out in the right, humid end and go to the dry end. Readings are in 2 parts as indicated. See text.

Elevated humidity, see Figure 6. Distilled water (100 µl) was placed into a 2.5×20 cm test tube left vertically overnight with a Parafilm cover. Water at the bottom of the tube was then pipetted out. After 30 minutes of allowing the humidity in the covered tube to equilibrate, about 20 flies were placed inside. The humidity in a same but vacant tube, measured with a humidity monitor (Traceable Hygrometer), was 85 to 90%. A tube of room humidity measured at 10 to 15% (“dry”), was also Parafilmed. (Drierite could be used to lower the humidity.) Both tubes were then placed into a dark room for 5 minutes, 15 cm away from and parallel to a visible-light source turned to face away from the flies. At 5 minutes the tube of flies was briefly oriented towards the light to gather the flies at the closed end. Then the humid and dry tubes were connected. The number of flies was then counted every minute for each quarter of the two tubes for 20 minutes. In a control there was no humidity gradient.

#### E. Response to temperature: thermotaxis

**Figure 7:**
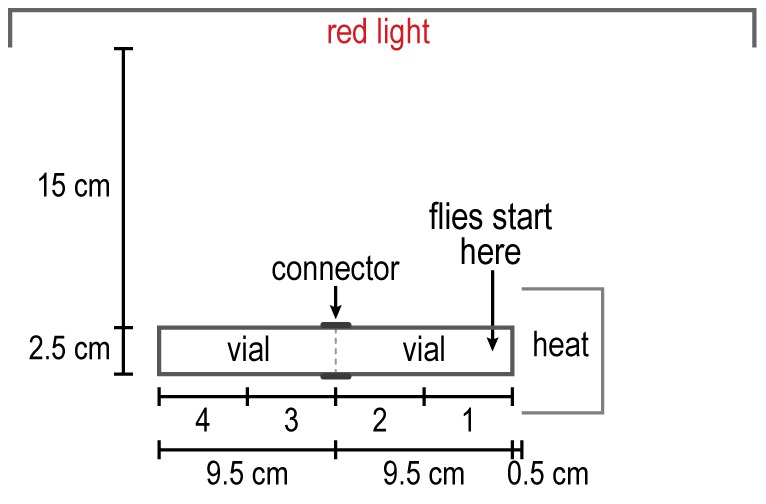
How to measure response to heat. Flies start at the right, warm end and go to the cool end. Readings are in 4 parts as indicated. See text.

Increasing temperature, see Figure 7. The heat assay was performed in a dark room. The apparatus, consisting of two vials, each 2.5×9.5 cm, put together by means of a connector, was placed 0.5 cm from a hotplate (Barnstead Thermoline Type 1200) on its side and set at about 150°C. A stable heat gradient was established in the apparatus in 20 to 30 min. A parallel tube containing the probe of a thermometer (indoor-outdoor, RadioShack) was placed adjacent to the apparatus to measure the temperature of the air at various places in the parallel tube. Flies to be tested were put into a vial plugged with cotton and left in the dark for 30 min. The flies were then inserted into the apparatus by separating the two vials, putting the flies into the warm half, and pounding them to the warm end, then reconnecting the vials, while an overhead red light (poorly visible by flies) was turned on briefly to provide visibility. Finally, the number of flies in each four compartments was counted every 5 minutes while briefly engaging the red light each time. In a control there was no temperature gradient.

**Figure 8:**
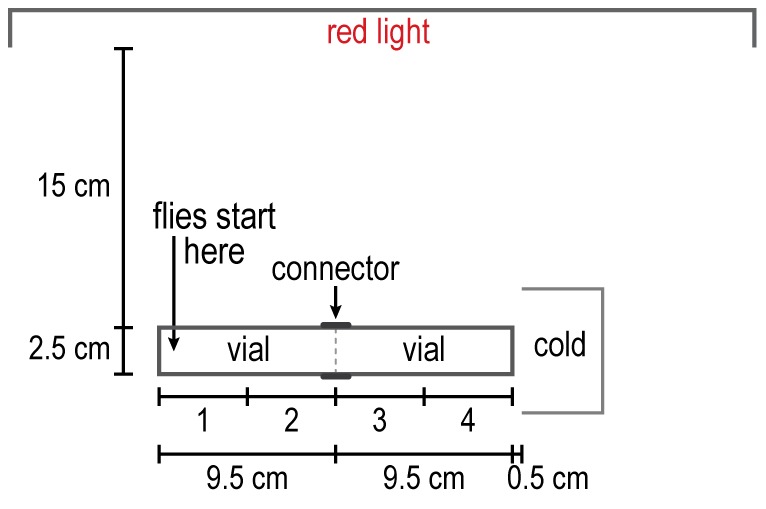
How to measure response to cold. Flies start out at the left end and avoid going to the cold end. Readings are in 4 parts as indicated. See text.

Decreasing temperature, see Figure 8. The cold assays were performed just like the heat assays (Figure 7) except that a beaker of ice in direct contact with the apparatus replaced the hotplate, and the flies started out at the room-temperature end. In a control there was no temperature gradient.

#### F. Response to gravity: geotaxis (also called gravitaxis)

**Figure 9:**
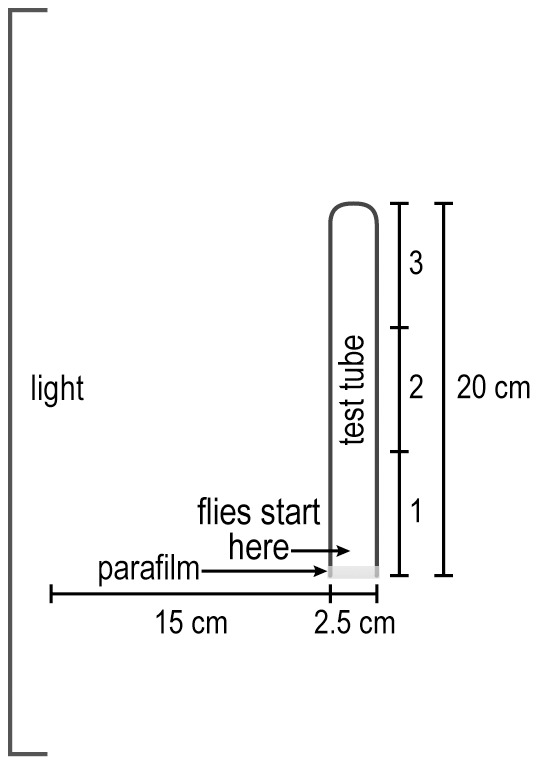
How to measure response to gravity. Flies start in the bottom of a vertical test tube, then migrate to the top. Readings are in 3 parts as indicated. See text.

Effect of gravity, see Figure 9. In a dark room containing a 2.5×20 cm test tube, about 20 flies were placed into a vial and then its open end was covered with cotton. The vial was placed horizontally and parallel to a light source 15 cm away for 30 minutes. Then the flies were gently pounded into the test tube held vertically, the tube was covered with Parafilm, then the tube was turned upside down, and the flies were gently pounded down to the Parafilm. At the same time the light source was placed vertically. Then the number of flies in each third of the tube was counted every minute. A horizontal control was used. We also tried a medium-diameter tube (4.5×20 cm) and a yet larger diameter tube (a graduated cylinder 10.25×20 cm).

### II. Use of these methods to isolate unresponsive but motile mutants

Male flies were mutagenized overnight with 25 mM ethyl methanesulfonate in 1% sucrose on Kimwipe paper. Then they were mated with virgin attached-X females, then at six days the flies were removed, and at around two weeks the new flies were studied (approximately 1 to 7 days old). About 5,300 of these F1 males were screened at 34°C, around 500 at a time, by placing them near the repellents benzaldehyde and heat at one end of an 11×70 cm tube while the other end had the attractants light and a favored temperature. Parental flies quickly migrated to the attractant end, while mutant flies were distributed throughout. In this way five independent male mutants were isolated (see RESULTS, Part II). Full details of these methods will be presented by Vang *et al.* in “*Drosophila* mutants that are motile but fail to respond to stimuli” (manuscript in preparation, 2012).

## Results

### I. Methods to measure responses

#### A. Response to light: phototaxis

**Figure 10:**
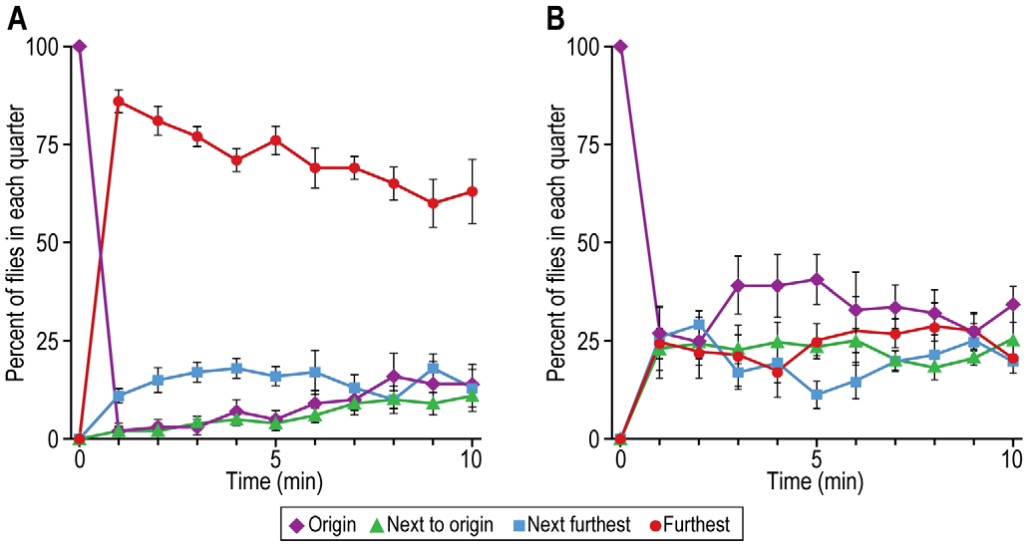
Attraction of flies to light. A) Light-deprived flies are presented with light. Data are shown for each of the 4 parts of the assay. Mean ± S.E.M. for 5 experiments. B) Control with parallel light. Mean ± S.E.M. for 6 experiments.

Attraction to light. Flies were placed away from a perpendicular light source, see Figure 1 for procedure. Figure 10A shows that the flies were attracted to the light initially, then the response began to decrease (see Discussion). When the light source was parallel to the flies, so that there was no light gradient for them, they distributed randomly (Figure 10B).

#### B. Response to volatile chemicals: smell chemotaxis, olfaction

**Figure 11:**
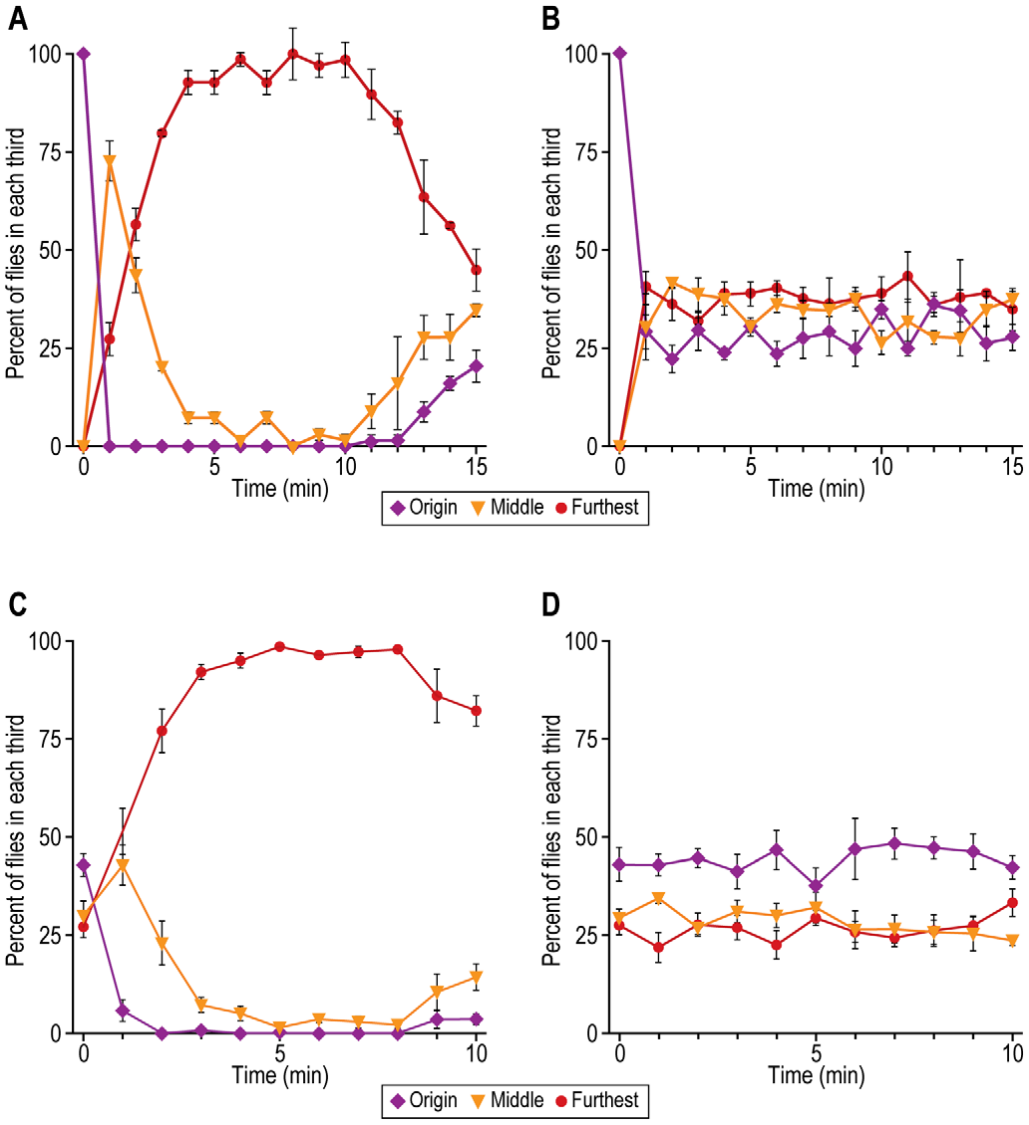
Repulsion of flies by a volatile chemical, benzaldehyde. Method 1: A) Flies begin near benzaldehyde at start. Data are presented for each of the 3 parts of the assay. Mean ± S.E.M. for 3 experiments. B) Control without benzaldehyde. Mean ± S.E.M. for 3 experiments. Method 2: C) Flies randomly distributed at start. Data are presented for each of the 3 parts of the assay. Mean ± S.E.M. for 7 experiments. D) Control without benzaldehyde. Mean ± S.E.M. for 6 experiments.

Repulsion by benzaldehyde. Method 1 (Figure 11A): Flies were placed near the benzaldehyde (see Figure 2A for procedure). Figure 11A shows that at 100 mM benzaldehyde the flies were repelled. By 10 min, the repulsion began to decline, presumably because further diffusion resulted in a benzaldehyde gradient that became too shallow for the flies to find a more attractive concentration and move towards it. With no addition of benzaldehyde, the flies became randomly distributed (Figure 11B).

Method 2 (Figure 11C): Flies were randomly distributed at the start (see Figure 2B for procedure). Figure 11C shows that at 100 mM benzaldehyde the flies were repelled by 10 minutes; at longer times (data not shown) the flies began to be again randomized, presumably because the benzaldehyde then started to become close to randomly distributed. With no addition of benzaldehyde (Figure 11D) the flies remained randomly distributed.

#### C. Response to nonvolatile chemicals: taste chemotaxis

**Figure 12:**
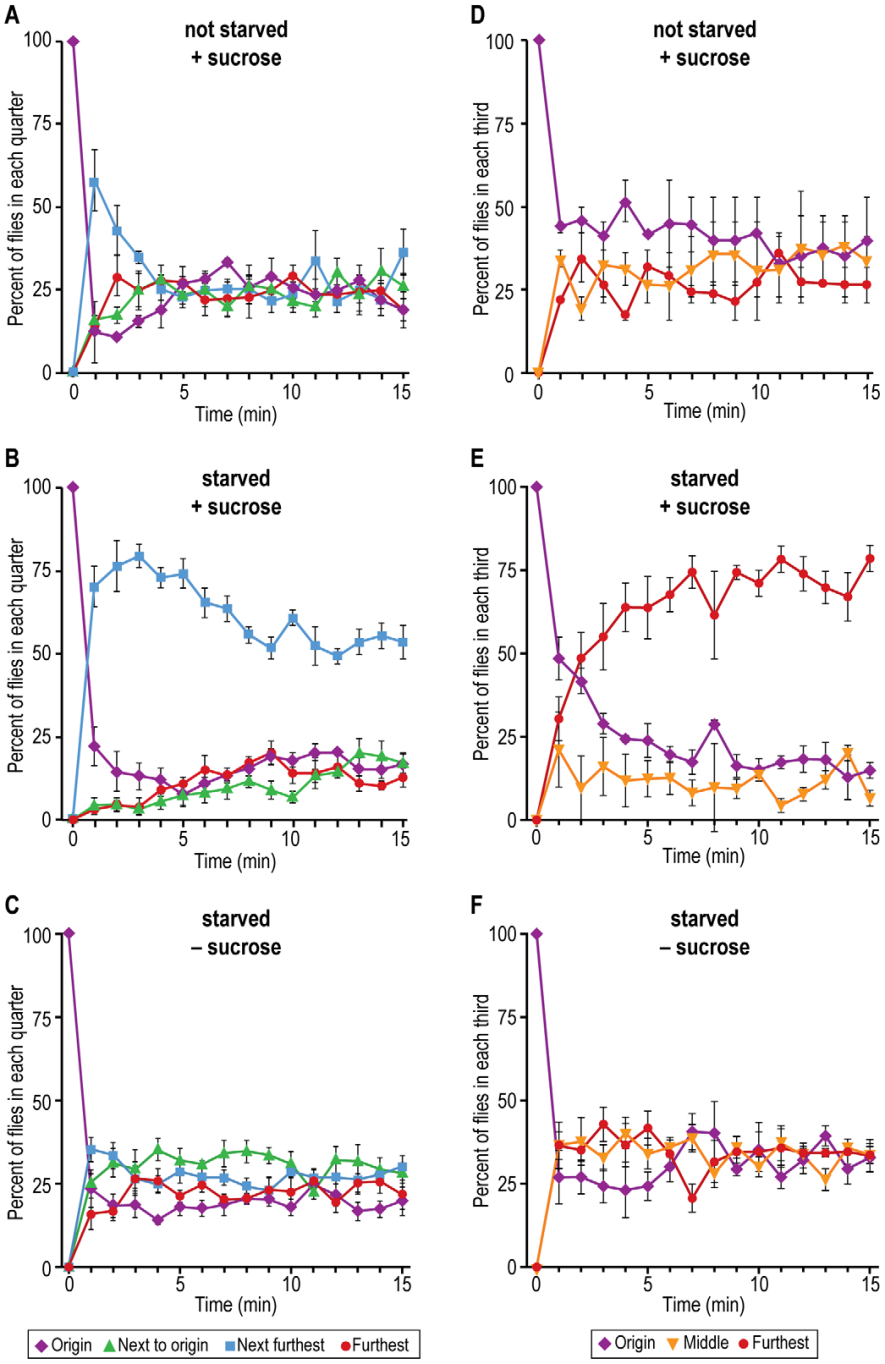
Attraction of flies to a non-volatile chemical, sucrose. Method 1: Agar throughout, sucrose in one half. Data are presented for each of the quarters. A) Flies not starved. Mean ± S.E.M. for 3 experiments. B) Flies starved. Mean ± S.E.M. for 5 experiments. C) Flies starved, control without sucrose. Mean ± S.E.M. for 5 experiments. Method 2: Sucrose with agar at end. Data are presented for each of the thirds. D) Flies not starved. Mean ± S.E.M. for 2 experiments. E) Flies starved. Mean ± S.E.M. for 4 experiments. F) Flies starved, control without sucrose. Mean ± S.E.M. for 4 experiments.

Attraction to sucrose. Method 1: Half the apparatus had 100 mM sucrose and half did not, and there was agar throughout; then either not starved or starved flies were placed into the non-sucrose end furthest away from the sucrose (see Figure 3A for procedure). Figure 12A shows that initially the unstarved flies were weakly attracted but not soon thereafter. Figure 12B shows that the starved flies departed from the non-sucrose end to go into the first sucrose compartment. (Since sucrose is not volatile, they must get there by random motility.) They stayed there presumably until satiated with sucrose, without going on into the next sucrose compartment. With no sucrose, the starved flies randomized (Figure 12C).

Method 2: In an alternative assay the 100 mM sucrose was placed in 1 ml of agar furthest away from the starved flies with no agar throughout (see Figure 3B for procedure). Figure 12D shows that unstarved flies did not accumulate at the sucrose. Starved flies accumulated near the sucrose (Figure 12E). With no sucrose, the starved flies distributed randomly (Figure 12E).

**Figure 13:**
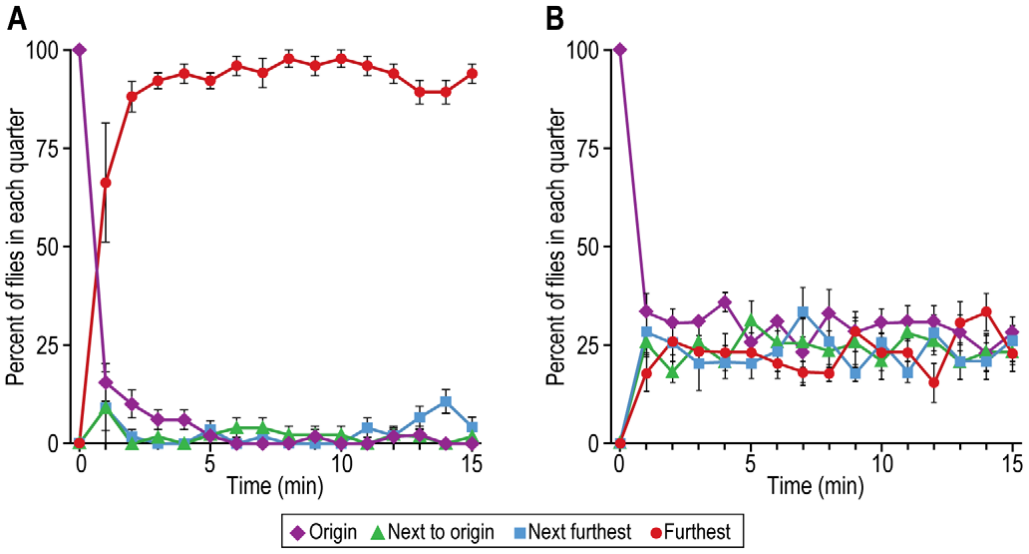
Attraction of flies to water. A) Water deprived flies are presented with water. Data are shown for each of the quarters. Mean ± S.E.M. for 5 experiments. B) Control without water. Mean ± S.E.M. for 4 experiments.

Attraction to water. Flies made thirsty overnight by keeping them in sucrose crystals without water were placed furthest away from 0.5 ml distilled water in Kimwipe (see Figure 4 for procedure). Then they were attracted to the water, see Figure 13A. With no addition of water the flies distributed randomly already by 1 minute, including at the end where water would be in the experimental setup (Figure 13B); this suggests that water may be tasted rather than smelled (see Discussion).

**Figure 14:**
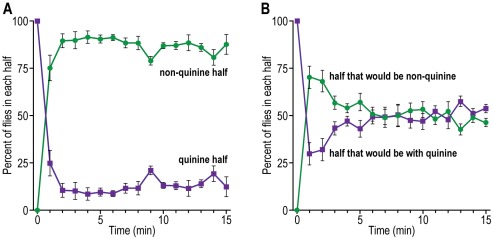
Repulsion of flies by a non-volatile chemical, quinine HCl. A) Flies are exposed to quinine. Data are presented for the quinine half and the half lacking quinine. Mean ± S.E.M. for 5 experiments. B) Control without quinine HCl. Mean ± S.E.M. for 6 experiments.

Repulsion by quinine HCl. Half the apparatus had 100 mM quinine HCl and half did not; then flies were pounded into the quinine half at its end furthest away from the non-quinine half (see Figure 5 for procedure). Figure 14A shows that the flies left the quinine half to accumulate in the non-quinine half. With no quinine, the flies randomized (Figure 14B).

#### D. Response to humidity: hygrotaxis

**Figure 15:**
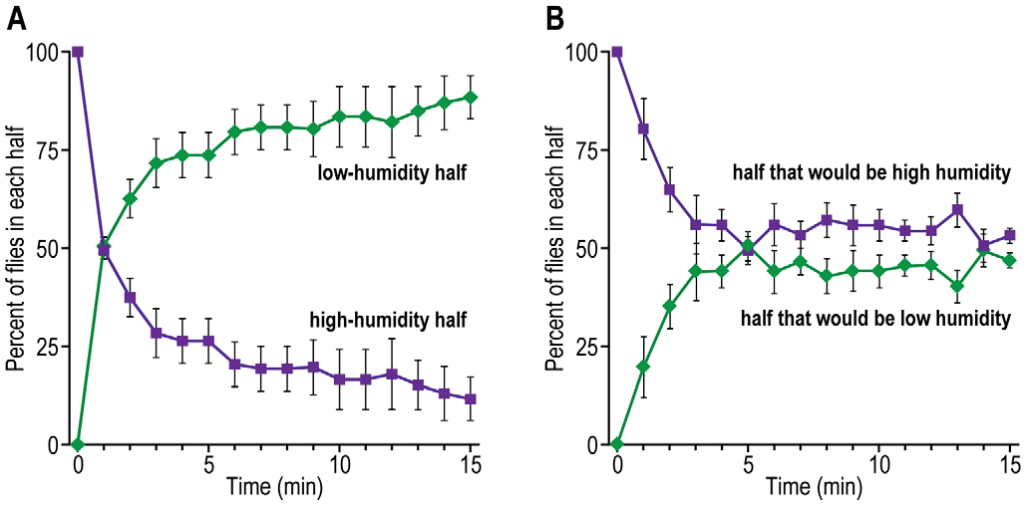
Repulsion of flies by high humidity. A) Flies are exposed to high humidity. Data are presented for each half. Mean ± S.E.M. for 3 experiments. B) Control without high humidity. Mean ± S.E.M. for 4 experiments.

Repulsion by high humidity. Flies were placed into high (85–90%) humidity (see Figure 6 for procedure). Then they moved to low (10–15%) humidity, as shown in Figure 15A. Without a humidity gradient the flies distributed randomly (Figure 15B).

#### E. Response to temperature: thermotaxis

**Figure 16:**
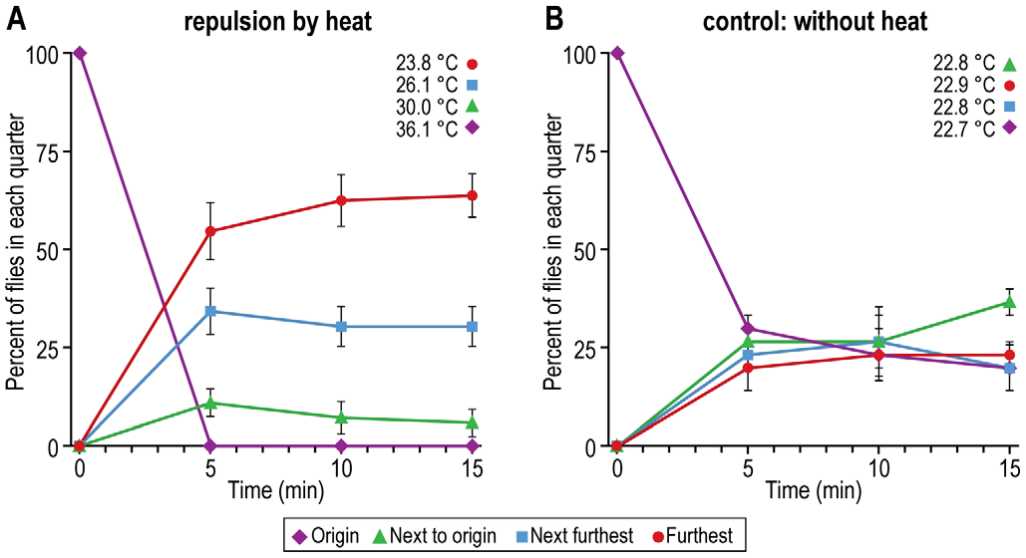
Repulsion of flies by heat. A) Flies are placed near a hot plate. Data from each of the quarters are presented for every 5 minutes. Mean ± S.E.M. for 4 experiments. B) Control without heat. Mean ± S.E.M. for 2 experiments. Temperatures in each quarter are the average of 4 experiments for A and 2 experiments for B.

Repulsion by high temperature. A hot plate generated a heat gradient in the apparatus (see Figure 7 for procedure). Flies placed in the hottest part ran away from there into cooler portions near the room-temperature end, as shown in Figure 16A. Without the heat gradient the flies distributed randomly (Figure 16B).

**Figure 17:**
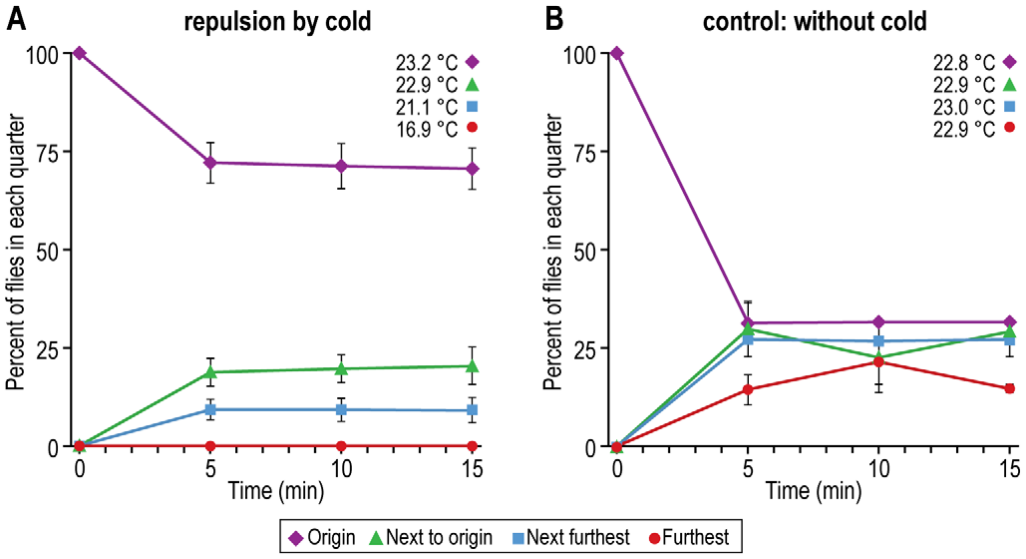
Repulsion of flies by cold. A) Flies are placed opposite a cold source. Data from each of the quarters are presented for every 5 minutes. Mean ± S.E.M. for 8 experiments. B) Control without cold. Mean ± S.E.M. for 2 experiments. Temperatures in each quarter are the average of 8 experiments for A and 2 experiments for B.

Repulsion by low temperature. Ice generated a cold gradient in the apparatus (see Figure 8 for procedure). Figure 17A shows that flies starting out at the room-temperature end avoided going to the colder regions. Without the cold gradient, the flies distributed randomly (Figure 17B).

#### F. Response to gravity: geotaxis (also called gravitaxis)

**Figure 18:**
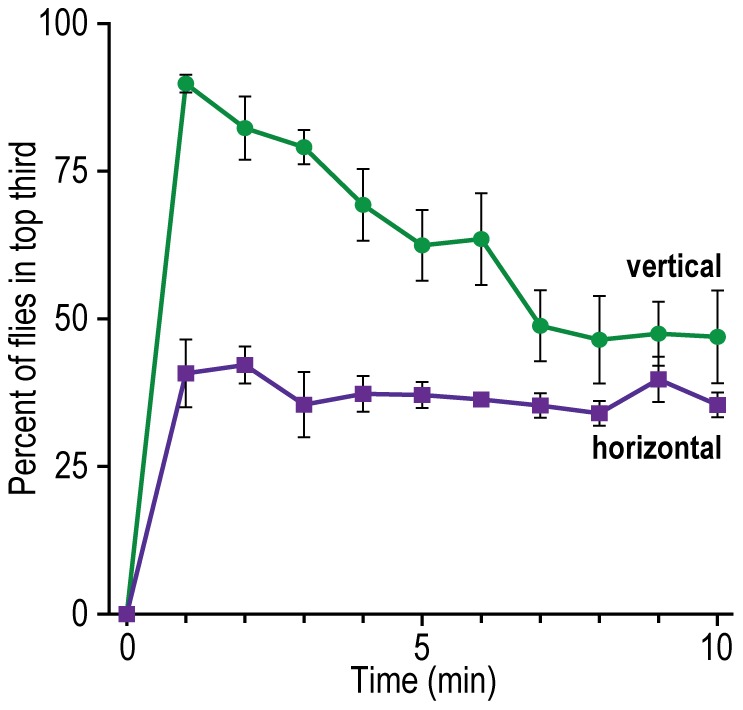
Repulsion of flies by gravity. Data are presented for the top third of the tube. Mean ± S.E.M. for 6 vertical experiments and 6 horizontal experiments.

Repulsion by gravity. Flies were subjected to a gravity gradient by placing them at the bottom of a vertical tube (see Figure 9 for procedure). Figure 18 tells that flies go up the vertical tube. They go up along the wall. This tube was 2.5 cm wide. Fewer flies did that when the width was increased to 4.5 cm (7 experiments, data not shown) and still fewer when increased to 10 cm (4 experiments, data not shown), presumably because then fewer of the flies have access to the wall. In a horizontal 2.5 cm tube the flies distributed randomly (Figure 18).

### II. Use of these methods to describe an unresponsive but motile mutant

**Figure 19:**
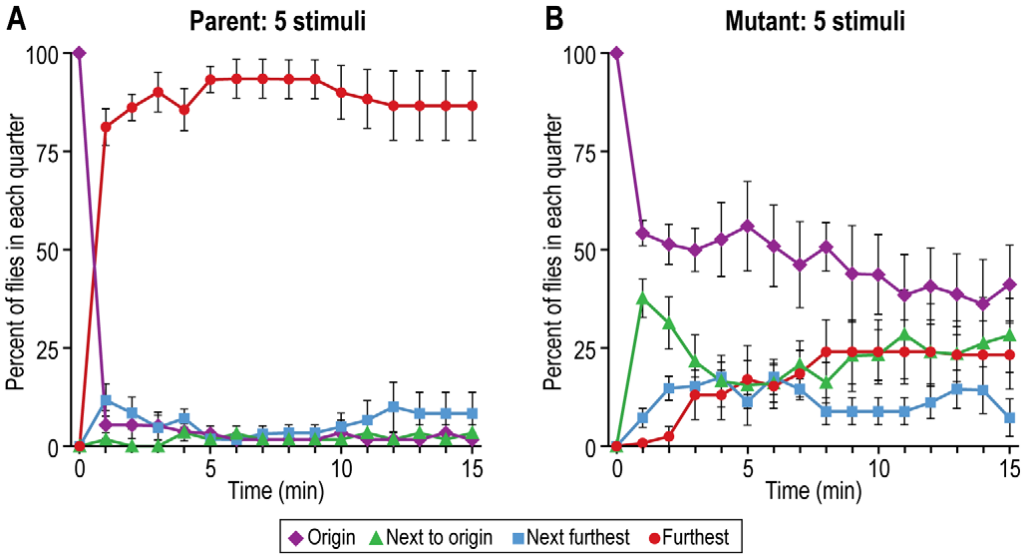
A mutant that fails to respond to multiple stimuli. At the right end of an 11 cm×70 cm tube were placed two repellents—benzaldehyde and high temperature (38°C)—and at the left end two attractants—light and a favored temperature (29°C). Flies were placed near the repellent end (labeled here “origin”). A) Nearly all the parental flies went to the attractant end (labeled here “furthest”). Mean ± S.E.M. for 7 experiments. B) Mutant flies failed to be repelled or attracted. Mean ± S.E.M. for 4 experiments. Details will be presented elsewhere [Vang *et al.*, “*Drosophila* mutants that are motile but fail to respond to stimuli” (manuscript in preparation, 2012)].

Flies were placed in a tube near one end that had the repellents benzaldehyde and heat, while the other end had the attractants light and a favored temperature (see MATERIALS AND METHODS, Part II). By one minute the parent had already moved away from the end that had repellents to the end that had attractants, while the mutants failed to do that or did it slowly. Figure 19A shows the response of parent and Figure 19B of a mutant (four other mutants have also been studied). When only a single stimulus was presented, the parent by one minute had already responded, while the mutant did not respond; such results were obtained as well for the following single stimuli (nearly all those described above): light, benzaldehyde, quinine, temperature, and gravity (data not shown). In the absence of any stimuli the mutants moved slightly slower than the parent. Motility was also judged by a walking assay which continuously records freely walking single flies at 22°C in a tube that has an infrared light gate situated in the middle of its long axis; interruption of the light gate triggers a circuit [19]. Again the mutants moved slightly slower than the parent (data not shown). All the above comes from experiments at 34°C, but at room temperature the same results were obtained as far as carried out. Full details of all these results will be presented by Vang *et al.* in “*Drosophila* mutants that are motile but fail to respond to stimuli” (manuscript in preparation, 2012). A genetic study of the mutants is underway.

## Discussion

### I. Methods to measure responses

In this report we demonstrate the responses of *Drosophila* to a variety of stimuli by use of methods that are easy to carry out. Earlier work by others has often used different approaches that are sometimes more complex than these. Here we summarize some of that earlier work and relate it to the methods described in this report.

The response to light by fruit flies was measured already in 1905 [20] and then in 1918 [21] by methods basically similar to the one we employed although ours is perhaps “simpler” (see Figure 10A here). In another assay similar to the one here, fruit flies in a tube were separated from an empty tube by means of a barrier that could be removed when the light was turned on [22], Figure 3 in ref. [23]. Similarly the effect of age of the flies on phototaxis was measured in tubes that had the flies in a distant dark portion while light shone in at the non-dark portion [24]. The response of fruit flies to light has been measured in a countercurrent apparatus [25].

“Fast” phototaxis is movement towards light by flies that have been shaken, while movement towards light in absence of shaking is “slow” phototaxis [26]. To prepare the flies for study in Figure 10A they were first gently pounded down, so the initial response could be “fast” phototaxis; after some minutes the response shown in Figure 10A slows down, which may be due to “slow” phototaxis, since the flies were then no longer being shaken. Alternatively, the slow-down could be due to slow adaptation, which operates on a time scale of many minutes, instead of fast adaptation, which occurs within a second [3], [27].

Methods for measuring the behavioral response of fruit flies to volatile chemicals – smell chemotaxis, olfaction – have been reviewed [28]. In an avoidance assay flies in a test tube run away from repellent placed at the open end on a Q-tip coming out of a cotton swab [29]. The assay used in the present report is fundamentally similar to that (Figures 11A and 11C). In the jump assay a single fly jumps when a pulse of odorant is passed over it [30]. Another test for olfaction is based on a single tethered fly turning toward a higher concentration of chemical [31]. In the Y-shaped assay [32] and the T-shaped assay [33]–[35] the flies avoid or are attracted into the branch that contains odorant. Response to volatile attractants has been measured in a 1- to 2-day assay by relying on diffusion of odor coming out of a micropipette [36]. We have tried, but did not succeed, to find a faster assay for volatile attractants.

For assays of the behavioral response to non-volatile chemicals – taste chemotaxis – see a review of available methods [28]. Flies have been fed on different chemicals or food sources each colored with a different dye, then color ingested was measured to determine the amount of tastant eaten [37]–[39]. The proboscis extension reflex has been used to measure a variety of repulsive or attractive tastants [40]–[43]. The methods used to assay for response to sucrose (Figures 12A and 12C) and water (Figure 13A) and response to quinine (Figure 14A) are different from those methods.

In the case of responding to water, the proboscis extension reflex has been used [42], [43] and the amount of water consumed by the fly has been measured [43]. The present assay for response to water (Figure 13A) is different from these: flies had to find the water. How did they do that? In a control that had no water at its end, some flies did get to the end just as fast as when there was water at the end, so it is not necessary to invoke the idea that the flies had to smell the water in order to get to the water, although that remains a possibility.

To assay response to humidity, flies have previously been placed in the middle between moist air in one tube and dry air in another, then the flies made a choice [44]. Our assay for response to high humidity is basically similar although “simpler” (Figure 15A).

The response of flies to temperature has previously been measured by placing them in the middle between high temperature in one tube and low temperature in another, as above for humidity, and also by providing them with a linear thermal gradient [44]. A fly was shown to avoid heat by jumping when its abdomen was heated or when it was put on a hot plate [45]. Our assay demonstrates avoidance of heat (Figure 16A) and of cold (Figure 17A) by measuring the movement of the flies in a thermal gradient.

The response to gravity has previously been measured by use of a vertical countercurrent apparatus [17], [46] and a vertical maze [14], [16], [47]–[50]. In addition, flies have been observed as they climbed up a vertical tube [15], [21], [24], [51], [52]; our method (Figure 18) is basically similar, yet “simple” and quantitative.

### II. Use of these methods to describe unresponsive but motile mutants

The methods described in this report have proved suitable for our present research on the isolation and study of mutants that fail to be attracted or repelled although they are motile. Two repellents - benzaldehyde and heat - were placed at one end of a tube and two attractants - light and a favored temperature - were placed at the other end. Then the flies were put near the repellent end. The parent quickly went to the attractant end (Figure 19A) while the mutant did not (Figure 19B). The mutant failed also to respond to any individual attractant or repellent. Similar results were obtained for four other mutants we isolated [Vang *et al.*, “*Drosophila* mutants that are motile but fail to respond to stimuli” (manuscript in preparation, 2012)]; see elsewhere [53] for a preliminary account of this.

What is the defect in the mutants reported here? We suppose that sensed information from all the different sensory receptors goes finally to central processing, which is the place that directs movement towards or away from stimuli. We consider that central processing is defective in these mutants so that they can no longer respond to stimuli, but motility still occurs. Central processing may well turn out to be located in the central complex.

In previous work by others [54]–[61], mutants defective in attraction to light were isolated, then those that failed were tested anatomically for structural defects in their central complex. Those with such structural defects were found to be defective in motility. These authors have studied how motility is regulated by the central complex, while we have studied attraction and repulsions in largely motile mutants. It seems likely that both approaches deal with properties of the central complex.

## References

[1] Borst A (2009). *Drosophila*'s view on insect vision.. Curr Biol.

[2] Katz B, Minke B (2009). *Drosophila* photoreceptors and signaling mechanisms.. Front Cell Neurosci.

[3] Hardie RC (2012). Phototransduction mechanisms in *Drosophila* microvillar photoreceptors.. WIREs Membr Transp Signal.

[4] Vosshall LB, Stocker RF (2007). Molecular architecture of smell and taste in *Drosophila*.. Annu Rev Neurosci.

[5] Su C-Y, Menuz K, Carlson JR (2009). Olfactory perception: receptors, cells, and circuits.. Cell.

[6] Benton R, Vannice KS, Gomez-Diaz C, Vosshall LB (2009). Variant ionotropic glutamate receptors as chemosensory receptors in *Drosophila*.. Cell.

[7] Touhara K, Vosshall LB (2009). Sensing odorants and pheromones with chemosensory receptors.. Annu Rev Physiol.

[8] Montell C (2009). A taste of the *Drosophila* gustatory receptors.. Curr Opin Neurobiol.

[9] Weiss LA, Dahanukar A, Kwon JY, Banerjee D, Carlson JR (2011). The molecular and cellular basis of bitter taste in *Drosophila*.. Neuron.

[10] Liu L, Li Y, Wang R, Yin C, Dong Q (2007). *Drosophila* hygrosensation requires the TRP channels water witch and nanchung.. Nature.

[11] McKemy DD (2007). Temperature sensing across species.. Eur J Physiol.

[12] Hamada FN, Rosenzweig M, Kang K, Pulver SR, Ghezzi A (2008). An internal thermal sensor controlling temperature preference in *Drosophila*.. Nature.

[13] Rosenzweig M, Kang K, Garrity P (2008). Distinct TRP channels are required for warm and cool avoidance in *Drosophila melanogaster*.. P Natl Acad Sci U S A.

[14] Kamikouchi A, Inagaki HK, Effertz T, Hendrich O, Fiala A (2009). The neural basis of *Drosophila* gravity-sensing and hearing.. Nature.

[15] Sun Y, Liu L, Ben-Shahar Y, Jacobs JS, Eberl DF (2009). TRPA channels distinguish gravity sensing from hearing in Johnston's organ.. Proc Natl Acad Sci U S A.

[16] Desroches CE, Busto M, Riedl CAL, Mackay TFC, Sokolowski MB (2010). Quantitative trait locus mapping of gravitaxis behaviour in *Drosophila melanogaster*.. Genet Res Camb.

[17] Inagaki HK, Kamikouchi A, Ito K (2009). Methods for quantifying simple gravity sensing in *Drosophila melanogaster*.. Nat Protoc.

[18] Kernan MJ (2007). Mechanotransduction and auditory transduction in *Drosophila*.. Eur J Physiol.

[19] Martin I-R, Ernst R, Heisenberg M (1999). Temporal pattern of locomotor activity in *Drosophila melanogaster*.. J Comp Physiol A.

[20] Carpenter FW (1905). The reactions of the pomace fly (*Drosophila ampelophila* loew) to light,gravity, and mechanical stimulation.. Amer Nat.

[21] McEwen RS (1918). The reactions to light and to gravity in *Drosophila* and its mutants.. J Exp Zool.

[22] Pak WL, Grossfield J, White NV (1969). Nonphototactc mutants in a study of vision of *Drosophila*.. Nature.

[23] Pak WL (2010). Why *Drosophila* to study phototransduction?. J Neurogenet.

[24] Leffelaar D, Grigliatti T (1984). Age-dependent behavior loss in adult *Drosophila melanogaster*.. Dev Genet.

[25] Benzer S (1967). Behavioral mutants of *Drosophila* isolated by countercurrent distribution.. Proc Natl Acad Sci U S A.

[26] Heisenberg M (1972). Comparative behavioral studies on two visual mutants of *Drosophila*.. J comp Physiol.

[27] Frechter S, Elia N, Tzarfaty V, Selinger Z, Minke B (2007). Translocation of Gq alpha mediates long-term adaptation in *Drosophila* photoreceptors.. J Neurosci.

[28] Devaud J-M (2003). Experimental studies of adult *Drosophila* chemosensory behavior.. Behav Proces.

[29] Anholt RRH, Fanara JJ, Fedorowicz GM, Ganguly I, Kulkarni NH (2001). Functional genomics of odor-guided behavior in *Drosophila melanogaster*.. Chem Senses.

[30] McKenna M, Monte P, Helfand SL, Woodard C, Carlson J (1989). A simple chemosensory response in *Drosophila* and the isolation of *acj* mutants in which it is affected.. Proc Natl Acad Sci U S A.

[31] Borst A, Heisenberg M (1982). Osmotropotaxis in *Drosophila melanogaster*.. J Comp Physiol A.

[32] Rodrigues V, Siddiqi O (1978). Genetic analysis of chemosensory pathway.. Proc Indian Acad Sci.

[33] Dudai Y, Jan Y-N, Byers D, Quinn WG, Benzer S (1976). *dunce*, a mutant of *Drosophila* deficient in learning.. Proc Natl Acad Sci USA.

[34] Tully T, Quinn WG (1985). Classical conditioning and retention in normal and mutant *Drosophila melanogaster*.. J Comp Physiol A.

[35] Helfand SL, Carlson JR (1989). Isolation and characterization of an olfactory mutant in *Drosophila* with a chemically specified defect.. Proc Natl Acad Sci USA.

[36] Woodard C, Huang T, Sun H, Helfand SL, Carlson J (1989). Genetic analysis of olfactory behavior in *Drosophila*: a new screen yields the *ota* mutants.. Genet Soc Am.

[37] Tanimura T, Isono K, Takamura T, Shimada I (1982). Genetic dimorphism in the taste sensitivity to trehalose in *Drosophila melanogaster*.. J Comp Physiol A.

[38] Ishimoto H, Tanimura T (2004). Molecular neurophysiology of taste in *Drosophila*.. Cell Mol Life Sci.

[39] Amrein H, Thorne N (2005). Gustatory perception and behavior in *Drosophila melanogaster*.. Curr Biol.

[40] Falk R, Atidia J (1975). Mutation affecting taste perception in *Drosophila melanogaster*.. Nature.

[41] Wang Z, Singhvi A, Kong P, Scott K (2004). Taste representations in the *Drosophila* brain.. Cell.

[42] Inoshita T, Tanimura T (2006). Cellular identification of water gustatory receptor neurons and their central projection pattern in *Drosophila*.. Proc Natl Acad Sci U S A.

[43] Cameron P, Hiroi M, Ngai J, Scott K (2010). The molecular basis for water taste in *Drosophila*.. Nature.

[44] Sayeed O, Benzer S (1996). Behavioral genetics of thermosensation and hygrosensation in *Drosophila*.. Proc Natl Acad Sci U S A.

[45] Xu SY, Cang CL, Liu XF, Peng YQ, Ye YZ (2006). Thermal nociception in adult *Drosophila*: behavioral characterization and the role of the painless gene.. Genes, Brain Behav.

[46] Tempel BL, Livingstone MS, Quinn WG (1984). Mutations in the dopa decarboxylase gene affect learning in *Drosophila*.. Proc Natl Acad Sci USA.

[47] Hirsch J (1959). Studies in experimental behavior genetics: II. individual differences in geotaxis as a function of chromosome variations in synthesized *Drosophila* populations.. J Comp Physiol Psychol.

[48] Toma DP, White KP, Hirsch J, Greenspan RJ (2002). Identification of genes involved in *Drosophila melanogaster* geotaxis, a complex behavioral trait.. Nature.

[49] Armstrong JD, Texada MJ, Munjaal R, Baker DA, Beckingham KM (2006). Gravitaxis in *Drosophila melanogaster*: a forward genetic screen.. Genes, Brain Behav.

[50] Strauss R, Heisenberg M (1993). A higher control center of locomotor behavior in the *Drosophila* brain.. J Neurosci.

[51] Bainton RJ, Tsai LT-Y, Singh CM, Moore MS, Neckameyer WS (2000). Dopamine modulates acute responses to cocaine, nicotine and ethanol in *Drosophila*.. Curr Biol.

[52] Baker DA, Beckingham KM, Armstrong JD (2007). Functional dissection of the neural substrates for gravitaxic maze behavior in *Drosophila melanogaster*.. J Comp Neurol.

[53] Adler J (2011). My life with nature.. Ann Rev Biochem.

[54] Heisenberg M, Schildberger K, Elsner N (1994). Central brain function in insects: Genetic studies on the mushroom bodies and central complex in *Drosophila*. Fortschritte der Zoologie.. Neural Basis of Behavioural Adaptations.

[55] Heisenberg M, Boehl K (1979). Isolation of anatomical brain mutants of *Drosophila* by histological means.. Z Naturforsch.

[56] Strauss R, Heisenberg M (1993). A higher control center of locomotor behavior in the *Drosophila* brain.. J Neurosci.

[57] Strauss R (1995). A screen for EMS-induced X-linked locomotor mutants in Drosophila melanogaster.. J Neurogenet.

[58] Strauss R, Burrows M, Matheson T, Newlund PL (1955). Stumbling flies: A screen for X-linked locomotor mutants identifies new genes involved in the control and execution of walking in *Drosophila melanogaster*.. Nervous Systems and Behavior. Proc 4th Int Congress Neuroethology.

[59] Martin J-R, Raabe T, Heisenberg M (1999). Central complex substructures are required for the maintenance of locomotor activity in *Drosophila melanogaster*.. J Comp Physiol A.

[60] Strauss R (2002). The central complex and the genetic dissection of locomotor behaviour.. Curr Opin Neurobiol.

[61] Poeck B, Triphan T, Neuser K, Strauss R (2008). Locomotor control by the central complex in *Drosophila* – An analysis of the *tay bridge* mutant.. Develop Neurobiol.

